# Human lung-derived mesenchymal stem cell-conditioned medium exerts in vitro antitumor effects in malignant pleural mesothelioma cell lines

**DOI:** 10.1186/s13287-016-0282-7

**Published:** 2016-02-09

**Authors:** Lourdes Cortes-Dericks, Laurene Froment, Gregor Kocher, Ralph A. Schmid

**Affiliations:** University Hospital Berne, Department of Clinical Research, Division of General Thoracic Surgery, Berne, Switzerland

**Keywords:** Malignant pleural mesothelioma, Human lung mesenchymal stem cell-conditioned medium, Soluble factors, Sphere-forming cells, Cisplatin

## Abstract

**Background:**

The soluble factors secreted by mesenchymal stem cells are thought to either support or inhibit tumor growth. Herein, we investigated whether the human lung-derived mesenchymal stem cell-conditioned medium (hlMSC-CM) exerts antitumor activity in malignant pleural mesothelioma cell lines H28, H2052 and Meso4.

**Methods:**

hlMSC-CM was collected from the human lung-derived mesenchymal stem cells. Inhibition of tumor cell growth was based on the reduction of cell viability and inhibition of cell proliferation using the XTT and BrdU assays, respectively. Elimination of tumor spheroids was assessed by the anchorage-independent sphere formation assay. The cytokine profile of hlMSC-CM was determined by a chemiluminescence-based cytokine array.

**Results:**

Our data showed that hlMSC-CM contains a broad range of soluble factors which include: cytokines, chemokines, hormones, growth and angiogenic factors, matrix metalloproteinases, metalloproteinase inhibitors and cell–cell mediator proteins. The 48- and 72-hour hlMSC-CM treatments of H28, H2052 and Meso4 cell lines elicited significant decreases in cell viability and inhibited cell proliferation. The 72-hour hlMSC-CM incubation of H28 cells completely eliminated the drug-resistant sphere-forming cells, which is more potent than twice the half maximal inhibitory concentration of cisplatin.

**Conclusions:**

Our findings indicate that the cell-free hlMSC-CM confers in vitro antitumor activities via soluble factors in the tested mesothelioma cells and, hence, may serve as a therapeutic tool to augment the current treatment strategies in malignant pleural mesothelioma.

**Electronic supplementary material:**

The online version of this article (doi:10.1186/s13287-016-0282-7) contains supplementary material, which is available to authorized users.

## Introduction

In contrast to the early paradigm of cell replacement and differentiation of mesenchymal stem cells (MSCs) as a therapeutic mechanism, reports are accumulating that cell secretions are responsible for their beneficial effects [1]. MSCs are known to secrete a wide range of bioactive molecules such as growth factors, cytokines and chemokines that regulate their biology in an autocrine or paracrine manner in accordance with the environmental niche [2]. It has been recognized that MSC activities are mediated by secreted biomolecules, highlighting the importance of using MSC-derived conditioned media (MSC-CM) in regenerative medicine as well as in cancer therapy. Clinically, the use of cell-free MSC-CM may represent a better therapeutic tool compared with stem cell-based therapy as the former is easier to prepare, maintain, and transport to appropriate sites, and may also have less complications related to issues on cell transplants [3].

When MSCs arrive at a tumor niche, they produce a variety of soluble factors that either positively or negatively influence tumor growth [4]. Several reports have accounted for the antitumorigenic capacity of MSCs. Khakoo et al. [5] have demonstrated that intravenously injected human MSCs (hMSCs) in a mouse model of sarcoma could potently inhibit tumor growth. Cell lysates or supernatants of Wharton’s jelly-derived MSCs have manifested a capacity to inhibit cell growth of breast cancer, ovarian tumor and osteosarcoma cell lines, indicating tumor inhibitory properties [6]. Although hMSCs have been demonstrated to suppress proliferation and induce apoptosis of SKMES-1 and A549 lung adenocarcinoma cells via some soluble factors [7], no current knowledge of hMSC-CM actions on malignant pleural mesothelioma (MPM) is known.

MPM is a highly aggressive, chemoresistant lung cancer with a median survival of <1 year after diagnosis [8, 9]. Thus, new therapeutic strategies are imperative to improve patient survival. Given that soluble factors secreted by MSCs are supposed to mediate beneficial effects, we investigated whether the cell-free hlMSC-CM exerts an antitumor capacity in three MPM cell lines.

## Materials and methods

### Cell culture

The H28 and H2052 mesothelioma cell lines (LCD Promochem, France) were cultured in RPMI 1640 (PAA, Austria) containing 10 % fetal bovine serum (FBS; PAA, Austria) and 1 % penicillin/streptomycin (Invitrogen, Switzerland). ACC-Meso-4 cell line was obtained from Riken Cell Bank, Resource No: RBRC-RCB2293 (Ibaraki, Japan) and cultured using the above-mentioned culture medium. Cells were maintained at 37 °C, 95 % humidity and 5 % CO_2_.

### Isolation and characterization of hlMSCs

We have previously identified human lung parenchyma mesenchymal stromal cells, which are referred as human lung-derived mesenchymal stem cells (hlMSCs) in this study. hlMSCs were isolated by using our established protocol as reported in our previous work [10], which includes immunophenotyping and in vitro mesenchymal trilineage differentiation into chondrocytes, adipocytes and osteocytes (see Additional file 1).

### Collection of hlMSC-CM

Conditioned medium (CM) was collected from hlMSCs at passage 3. Cells were cultured at a density of 5 × 10^4^ in 10 cm cell culture dish containing MCDB 201 supplemented with ITS, 1 % antibiotic/antimytotic reagent (Hyclone), 1 % FBS and 20 ng/μl endothelial growth factor at 37 °C in a humid atmosphere with 5 % CO_2_. Upon reaching 70–80 % confluency, cells were washed once with phosphate-buffered saline (PBS) and then re-incubated with a serum-free MCDB 201 medium for 24 hours. The CM (supernatant) was then collected and centrifuged at 1200 × g for 10 minutes to remove cell components and was sterilized by passing through a 0.2-μm cell filter (BD Biosciences, Switzerland). Aliquots were subjected to cytokine array analysis or frozen at −80 °C for future applications. hlMSC-CM was used as it was, without dilution or addition of serum in all of the experiments in this project.

### Cell proliferation and cell viability assays

The XTT and BrdU assays were performed to assess the effect of hlMSC-CM on cell viability and cell proliferation, respectively (see Additional file 2).

### Cytokine array assay

The cytokine profile of hlMSC-CM was determined using the Human Angiogenesis Antibody Array (see Additional file 3).

### Sphere formation

The sphere formation assay used to evaluate the formation of nonadherent multicellular spheroids is described in Additional file 4.

### Drug treatment

For drug treatment, cisplatin (diamminedichloroplatinum (II); Bristol Myers Squibb, Switzerland) was used. Cells were seeded at a density of 5 × 10^4^ cells/well in six-well culture dishes and incubated for 24 hours prior to 48- and 72-hour treatments with the half maximal inhibitory concentration (IC_50_) or twice the IC_50_ of cisplatin (twice the IC_50_ means 2× the concentration of the IC_50_ value). The specified concentration corresponds to the previously determined IC_50_ value of the three MPM cell lines: H28 (15 μM), H2052 (13 μM), and Meso4 (12 μM) [11]. Following the cisplatin treatments at 37 °C, cells were washed with PBS, trypsinized, and single-cell suspension was prepared for sphere formation assay.

### Statistical analysis

Data are reported as means ± standard deviations. For analysis of differences in multiple groups, one-way analysis of variance and Bonferroni’s post-hoc test were performed using Prism 6.0 (GraphPad Software, USA). A *p* value <0.05 was considered significant.

## Results

### hlMSC-CM contains soluble factors

**Fig. 1:**
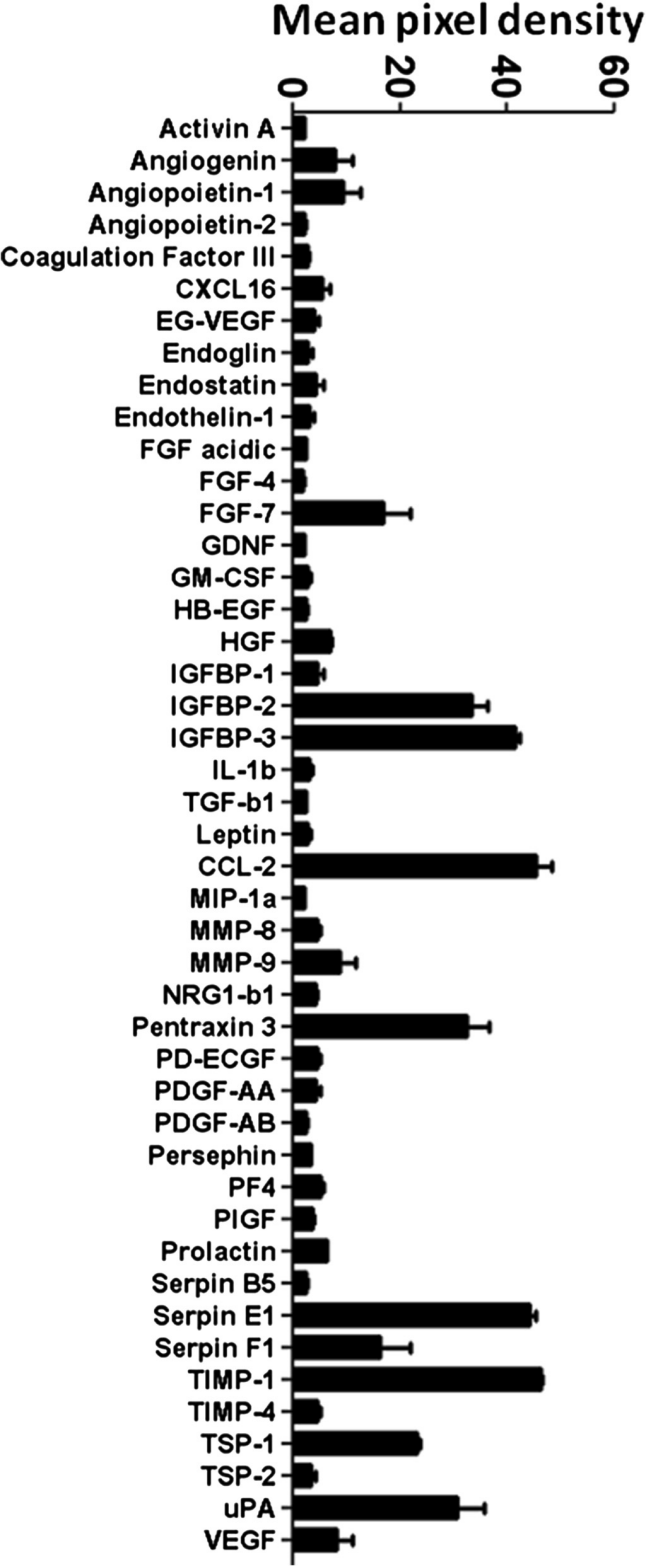
hlMSC-CM contains a broad range of soluble factors. The CM (supernatant) of hlMSCs grown for 24 hours in a culture medium without FBS was collected and subjected to a cytokine array assay as described in the Materials and methods. Results are representative of one of the three independent experiments

We have previously identified hlMSCs exhibiting plastic adherence, the immunophenotype and trilineage differentiation capacity consistent with the established features of MSCs [10, 12, 13]. We initially investigated whether our hlMSC-CM contains soluble factors. We therefore collected the CM of the hlMSCs grown for 24 hours in the absence of FBS and analyzed it using the cytokine array. hlMSC-CM contained a broad range of soluble factors which included: cytokines, chemokines, hormones, growth factors, neurotrophic factors, endocrine and angiogenic factors, matrix metalloproteinases (MMPs), metalloproteinase inhibitors (TIMPs) and cell–cell mediator proteins (Fig. 1).

### hlMSC-CM inhibits cell proliferation and reduces cell viability in three MPM cells lines

**Fig. 2:**
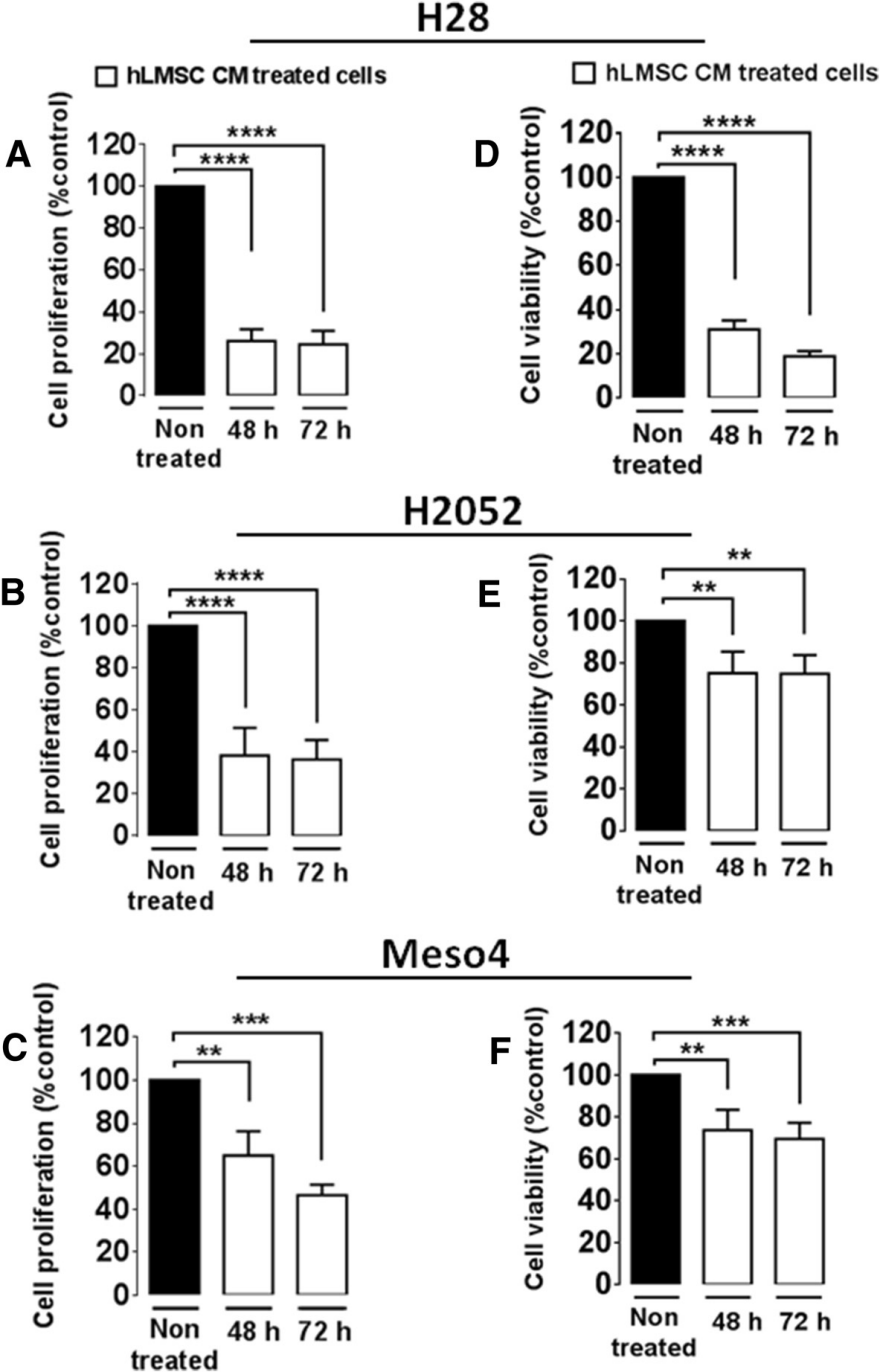
The inhibitory effect of hlMSC-CM on cell proliferation and reduction of cell viability in the three MPM cells lines. Significant inhibitions on cell proliferation of hlMSC-CM-treated H28 (**a**), H2052 (**b**) and Meso4 (**c**) cells were expressed as the percentage of proliferation relative to the nontreated (control) cells as determined by the BrdU assay. Reductions on cell viability in hlMSC-CM-treated H28 (**d**), H2052 (**e**) and Meso4 (**f**) cells were expressed as a percentage of cell viability relative to nontreated cells as evaluated by the XTT assay. Results are the means ± standard deviations of three independent experiments each. **p < 0.01, ***p < 0.001, ****p < 0.0001. *h* Hours, *hLMSC CM* Human lung-derived mesenchymal stem cell-conditioned media

We studied the effect of hlMSC-CM on the proliferative activity of H28, H2052 and Meso4 using the BrdU assay. The 48- and 72-hour treatments with hlMSC-CM elicited significant reductions in cell proliferation of H28 (48 hours –74 %; 72 hours –76 %), H2052 (48 hours –62 %; 72 hours –64 %) and Meso4 (48 hours –35 %; 72 hours –55 %) relative to the nontreated cells (Fig. 2a–c). We also investigated the effect of hlMSC-CM on cell viability after the treatment periods of 48 and 72 hours using the XTT assay. hlMSC-CM evoked significant reductions in cell viability in all tested cell lines: H28 (48 hours –69 %; 72 hours –81 %), H2052 (48 hours –25 %; 72 hours –25.3 %), Meso4 (48 hours –26.3 %; 72 hours –31 %) compared with the nontreated cells (Fig. 2d–f).

### hlMSC-CM eliminates sphere-forming phenotype in H28 cells

**Fig. 3:**
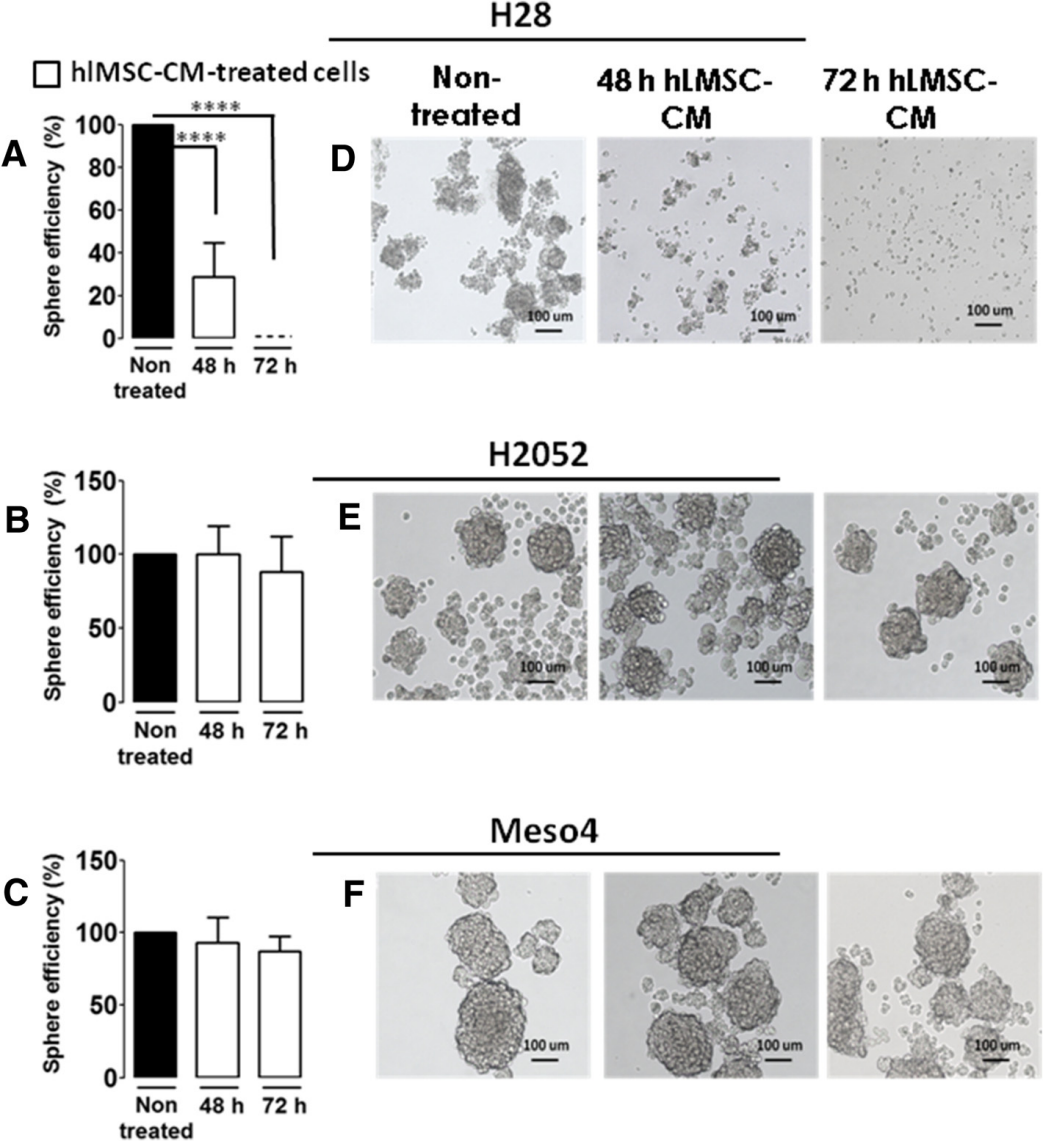
hlMSC-CM completely eliminates the sphere-forming cells in H28 cell line. Sphere-forming efficiencies of hlMSC-CM-treated cells were determined and compared with those of the nontreated H28 (**a**), H2052 (**b**) and Meso4 (**c**) cells. Results represent the means ± standard deviations of three experiments each. **d**-**f** Representative images of hlMSC-CM-treated and nontreated MPM cell lines as indicated. Images were taken with Leica DMI 4000B at 5× magnification. ****p < 0.0001. *h* Hours, *hlMSC-CM* Human lung-derived mesenchymal stem cell-conditioned media

In our previous work, we found cisplatin-resistant tumor spheres in H28, H2052 and Meso4 indicating the presence of putative cancer stem cells (CSCs), which may, in part, be responsible for drug resistance [11]. Hence, we investigated the ability of hlMSC-CM to eliminate these cells, and also compared its efficacy with cisplatin, a standard chemotherapy in the treatment of MPM [8, 9]. hlMSC-CM significantly reduced the sphere-forming efficiency by 70 % in H28 cells after 48 hours and, unexpectedly, fully eliminated them after the 72-hour treatment (Fig. 3a). These effects were not observed in H2052 and Meso4 cells (Fig. 3b and c). Representative images of the nontreated and hlMSC-CM-treated MPM cells are shown in Fig. 3d–f.

**Fig. 4:**
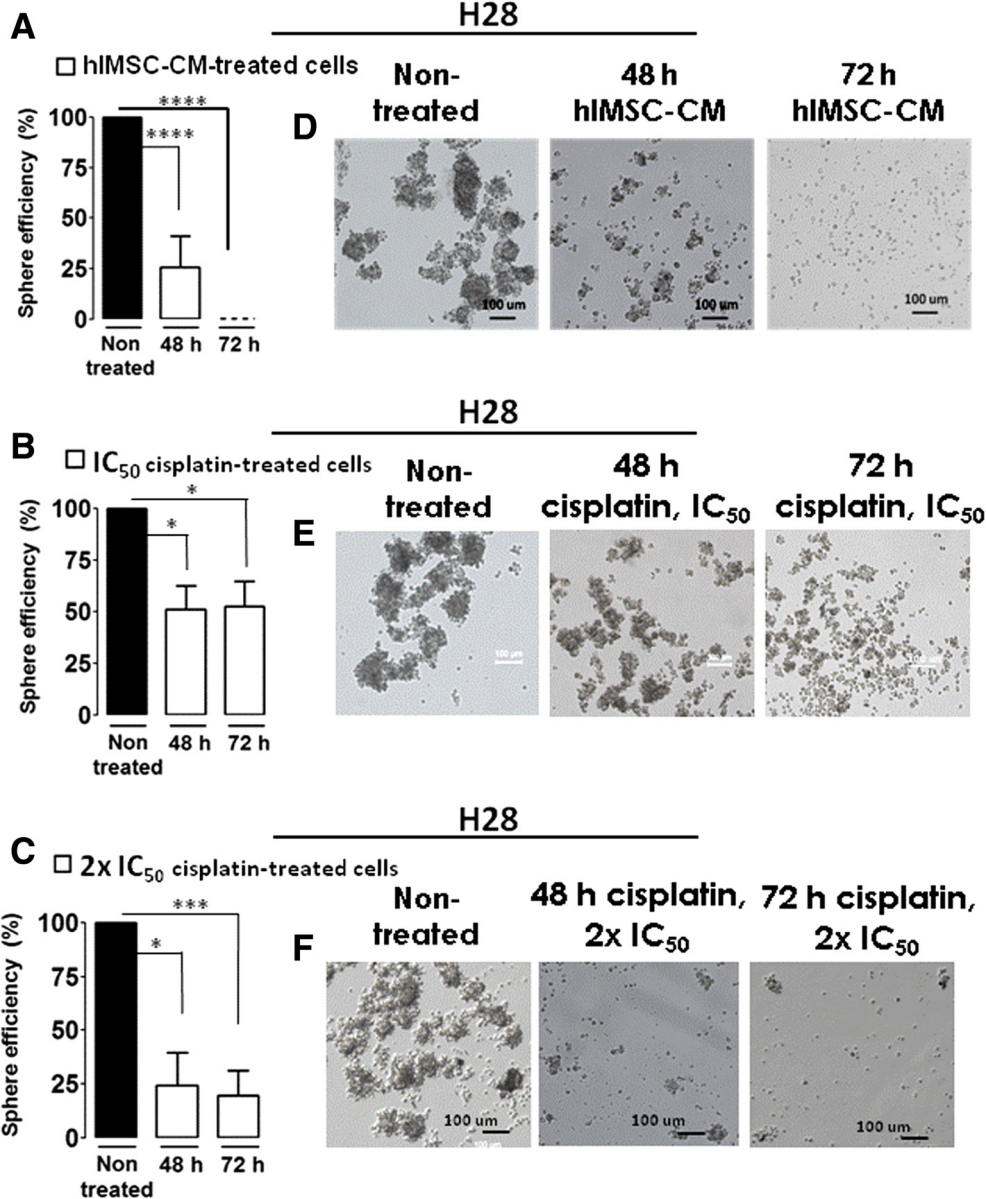
hlMSC-CM shows higher efficacy than twice the IC_50_ of cisplatin in inhibiting the growth of tumor spheres in H28 cells. H28 cells were treated in the absence or presence hlMSC-CM (**a**), 1× IC_50_ cisplatin (**b**) and 2× IC_50_ cisplatin (**c**) at the indicated periods after which sphere-formation assays were performed. Results indicate the sphere efficiencies of the hlMSC-CM-and cisplatin-treated cells relative to the nontreated controls. **d**-**f** Representative images of the hlMSC-CM-, cisplatin-treated cells and nontreated cells as indicated. Data represent the means and ± standard deviations of three independent experiments each. *p < 0.05, ***p < 0.001, ****p < 0.0001. *h* Hours, *hlMSC-CM* Human lung-derived mesenchymal stem cell-conditioned media, *IC*_*50*_ Half maximal inhibitory concentration

We then compared the inhibitory effect of hlMSC-CM on the growth of tumor spheres with that of IC_50_ and twice the IC_50_ of cisplatin in H28 cells. The 48-hour hlMSC-CM treatment significantly reduced the sphere-forming efficiency by 74 %, whereas the 72-hour incubation completely eliminated the tumor spheres (Fig. 4a). Using the IC_50_ of cisplatin, we obtained significant 50 % reductions in sphere efficiency after the 48- and 72-hour treatments (Fig. 4b), and significant decreases of 68 and 73 % after analogous incubation periods using twice the IC_50_ of cisplatin (Fig. 4c). Our results showed that hlMSC-CM is capable of fully eliminating the sphere-forming phenotype in H28 cells, and that this effect has a higher efficacy compared with twice the IC_50_ of cisplatin. Representative images of the nontreated and hlMSC-CM-treated H28 cells are shown in Fig. 4d–f.

## Discussion

One beneficial effect of MSCs is their inherent antitumor capacity—a current topic of investigation because of its future role in cancer therapy. Here, we report that hlMSC-CM contains a broad range of soluble factors, which conferred inhibition of cell proliferation and a decrease in cell viability in three MPM cell lines. Importantly, hlMSC-CM completely eliminated the cisplatin-resistant, sphere-forming cells.

We found a huge panel of cytokines, chemokines, growth, neurotrophic, endocrine and angiogenic factors, MMPs, TIMPs and cell–cell mediator proteins in our hlMSC-CM that elicited suppression of cell growth and reduced cell viability in the tested mesothelioma cells. These data substantiate the current paradigm that MSC-dependent beneficial effects are mediated primarily by secreted factors [1]. Evidences for the soluble factor-mediated antitumor capacity of hMSC-CM have been reported such as the reduction of SK-MES-1 and A549 lung cancer cell proliferation by a downregulation of vascular endothelial growth factor (VEGF) expression and by an increased apoptosis ratio compared with the non-hMSC-CM-treated cells [7]. The in vitro treatment of ovarian cancer cells with heat shocked adipose stem cell-derived CM and amniotic-derived stem cells have resulted in decreased tumor cell viability compared with controls by inducing greater nuclear condensation and growth cycle arrest of tumor cells. Cytokine array of the CM demonstrated that the observed antitumor effect was mediated by angiogenin, IGF-binding protein 4, NT-3 and chemokine ligand 18 [14]. In a coculture study of lung adenocarcinoma (LAC) cell lines and MSCs or MSC-CM, Wang et al. [15] have shown evidence that oncostatin M, a differentiation-promoting cytokine, mediated the MSC-dependent inhibition of tumorigenicity and activation of mesenchymal epithelial transition in LAC cells. Extracellular matrix components produced by human umbilical cord blood MSCs have also contributed to the growth arrest of metastatic cancer cells by an upregulation of tumor suppressor phosphatase and tensin homolog (PTEN) in the tumor cells [16]. The data in our cytokine array include factors that are likely involved in conferring antitumorigenic effects of MSCs such as angiogenin, VEGF, transforming growth factor-beta (TGF-b) and hepatocyte growth factor (HGF) [14, 17]. They also showed increased levels of biomolecules that have been linked to diverse roles in the malignant setting, which include IGFBP-1, IGFBP-2, CCL2, Pentraxin, SerpinE1, SerpinE2, TIMP-1 and uPA [18–23]. Further investigation is necessary to identify the specific factors that are implicated in the observed antitumorigenic property of hlMSC-CM in the tested mesothelioma cells.

MSCs have diverse effects on tumor growth. In contrast to the reported tumor-promoting capacity of MSCs, our findings showed a tumor-inhibiting effect of hlMSC-CM in the tested MPM cell lines. Tumor-promoting activity is exerted either by paracrine secretion of growth factors and antiapoptotic factors, or by differentiating into tumor-associated fibroblasts, which can enhance tumor growth, metastasis formation and therapy resistance [4]. Adversely, MSCs and/or MSC-derived CM also confer antitumorigenic responses as have been demonstrated by the inhibition of tumor growth in leukemia [24], prostate carcinoma [25], Kaposi’s sarcoma [5], colon carcinoma [26], breast cancer [27] and murine lung cancer [28] under in vitro and in vivo conditions. Diverse mechanisms have been reported to be involved in the antitumor potential of MSCs that include apoptosis caused by an upregulation of TRAIL, cell cycle arrest, blocking of the PI3K/AkT pathway, expression of tumor suppressor genes, downregulation of the Wnt pathway, expression of DKK1 and cytokine-mediated process. Operationally, MSCs have also been proven to home to tumor sites and exert suppression of tumor growth amongst others [29]. Our data point to a soluble factor-mediated antitumor effect of hlMSC-CM reflecting the notion that hMSCs have the innate capacity to produce biomolecules in response to the tumor microenvironment.

Sphere formation represents one of the basic features of CSCs, a rare subpopulation within a tumor, which is supposed to be a crucial player in the initiation, invasiveness and drug tolerance in different tumors including MPM [11, 30–35]. A striking property of hLMSC-CM observed in this study is its capacity to fully eliminate the sphere-forming phenotype in H28 cells. The failure of hlMSC-CM to abolish the sphere-forming cells in H2052 and Meso4 may be due to the differences in the histological composition of the tested cell lines. MPM is divided into three histological subtypes: epitheloid, sarcomatoid and biphasic [9]. Multicellular spheroids possess heterogeneity similar to that of tumor in vivo and their drug resilience has appeared to be analogous to the natural resistance observed in patient tumors [36]. Therefore, testing for sphere formation after drug treatment would represent a reliable method to evaluate chemoresistance, not only in vitro but also in an in vivo setting. Considering this, hlMSC-CM, which showed a high potency to eliminate tumor spheres, may be used to resolve drug tolerance in the epitheliod type of MPM as represented by the H28 cell line. Cisplatin is one of the standard components in the treatment of MPM, which as yet results in inadequate patient therapy [8]. Because the efficacy of hlMSC-CM to eliminate the sphere-forming cells in H28 is higher compared with twice the IC_50_ of cisplatin, it stands to reason that combining hlMSC-CM with conventional drugs may improve the current treatment modalities in MPM.

## Conclusion

Our study demonstrates that hlMSC-CM exerts in vitro antitumor effects in three MPM cell lines via soluble factors, which offers a potentially useful tool to augment the standard therapy in MPM.

## Additional files

Additional file 1:
**Isolation and characterization of hlMSCs.** (DOCX 16 kb) Additional file 2:
**Cell proliferation and cell viability assays.** (DOCX 11 kb) Additional file 3:
**Cytokine array assay.** (DOCX 10 kb) Additional file 4:
**Sphere formation.** (DOCX 10 kb)

## Abbreviations

CM: Conditioned media
CSC: Cancer stem cell
FBS: Fetal bovine serum
hlMSC: Human lung mesenchymal stem cell
hMSC: Human mesenchymal stem cell
IC_50_: Half maximal inhibitory concentration
LAC: Lung adenocarcinoma
MMP: Matrix metalloproteinases
MPM: Malignant pleural mesothelioma
MSC: Mesenchymal stem cell
PBS: Phosphate-buffered saline
TIMP: Metalloproteinase inhibitor
VEGF: Vascular endothelial growth factor
