# Supplementary material for: Human lung-derived mesenchymal stem cell-conditioned medium exerts in vitro antitumor effects in malignant pleural mesothelioma cell lines
Source: Stem Cell Res Ther. 2016 Feb 9;7:25. doi: 10.1186/s13287-016-0282-7 (PMC4748521; doi:10.1186/s13287-016-0282-7)
Supplement: Additional file 1: — Isolation and characterization of hlMSCs. (DOCX 16 kb) [file 13287_2016_282_MOESM1_ESM.docx]

**Isolation and characterization of hlMSCs**

Human lung mesenchymal stem cells (previously designated as human lung parenchyma mesenchymal stromal cells ) were isolated by using our established protocol as reported in our previous work [9]. In brief, lung biopsies collected in phosphate-buffered saline (PBS, Invitrogen, Switzerland), were cut into small pieces and washed in Hank`s balanced solution (Invitrogen) containing 2% penicillin/streptomycin, P/S (Invitrogen). The tissues were then treated with 0.1% protease type XIV (Sigma Aldrich, Switzerland) in Joklik`s minimum essential medium (JMEM, Sigma Aldrich) overnight at 4^o^C. Following this incubation, the tissues were transferred into a tube containing JMEM supplemented with 10% fetal bovine serum (FBS, Perbio Science, Switzerland) and were then pipetted repeatedly to release the pulmonary cells, which were subsequently filtered through a 100-µm cell strainer. The released pulmonary cells were washed and resuspended in MCDB 201 (Sigma Aldrich) containing insulin-transferrin-selenium supplements, ITS (Invitrogen) and 1% Pen/Strep. Cells were plated at a density of 33.333 cells/cm^2^ in 10 ml of culture medium onto a T_75_ culture flasks coated with Collagen 1 (10µg/cm^2^) (BD Biosciences, Switzerland). After 24 h of incubation at 37^o^C and 5% CO2, the cells were washed with MCDB-201 medium to remove the non-adherent cells, and were further incubated with MCDB-201 supplemented with ITS and 1% epidermal growth factor, EGF (Invitrogen) and 1% FBS to maintain the adherent hlMSC culture.

The immunophenotype was determined by flow cytometry-based analysis which included the markers: CD73, CD166, CD105, CD90, STRO-1 CD34, CD45, CD19, HLA-DR, CXCR4 and corresponding isotype controls (BD Biosciences). An indirect staining protocol was used. 0.5 - 1 x10^6^ cells were stained with 1 µg of primary antibody or appropriate isotype control for 30 min on ice. The cells were restained with Alexafluor 488-conjugated secondary antibody (1:500) (Invitrogen) for 30 min followed by two washes with PBS. Cells were resuspended in 300 µl of PBS prior to data acquisition using LSR II flow cytometer (BD Biosciences).

In vitro mesenchymal trilineage differentiation into chondrocytes, adipocytes and osteocytes was performed as follows. Cells were seeded at a density of 2.5 x 10^4^ on a six-well plate and were allowed to grow until 80% confluency in standard growth medium prior to exposure to different differentiation media (adipogenic, osteogenic and chondrogenic, [Hyclone, Belgium]) supplemented with stem cell growth serum (Hyclone) according to manufacturer`s instructions. Osteogenic differentiation was evaluated by BCIP/NTP staining for alkaline phophatase activity viewed under light microscopy. Adipogenic differentiation was based on accumulation of lipid droplets within the cells as determined by oil red staining viewed under phase-contrast microscopy. Chondrogenic differentiation was assessed by the presence of chondroitin as determined by toluidine blue staining visualized under light microscopy.
