# Supplementary material for: Human lung-derived mesenchymal stem cell-conditioned medium exerts in vitro antitumor effects in malignant pleural mesothelioma cell lines
Source: Stem Cell Res Ther. 2016 Feb 9;7:25. doi: 10.1186/s13287-016-0282-7 (PMC4748521; doi:10.1186/s13287-016-0282-7)
Supplement: Additional file 2: — Cell proliferation and cell viability assays. (DOCX 11 kb) [file 13287_2016_282_MOESM2_ESM.docx]

**Cell proliferation and cell viability assays**

The XTT (tetrazolium derivative) assay (Roche Chemical, Switzerland) was performed to evaluate the effect of hlMSC-CM on cell viability. Cells were plated at a density of 2 x 10^3^ cells/well in a 96-well plate and were incubated in the presence or absence of hlMSC-CM. Control cells (non-hlMSC-CM-treated cells were incubated in serum-free RPMI 1640). Following the 48 and 72 h incubation periods, XTT labeling mixture was added according to manufacturer`s instructions. Formazan production was measured spectrophotometrically with TECAN infinite M1000 at 450 nm. The BrdU (5-bromo-2`-deoxyuridine) assay was done to assess the effect of hlMSC-CM on cell proliferation. Cells were plated and treated with or without hlMSC-CM as described above. Five hours before the end of the incubation periods, BrdU reagent was added according to manufacturer`s procedures. Results were measured spectrophotometrically with TECAN infinite M1000 set at 450 nm.
