# Supplementary material for: Human lung-derived mesenchymal stem cell-conditioned medium exerts in vitro antitumor effects in malignant pleural mesothelioma cell lines
Source: Stem Cell Res Ther. 2016 Feb 9;7:25. doi: 10.1186/s13287-016-0282-7 (PMC4748521; doi:10.1186/s13287-016-0282-7)
Supplement: Additional file 3: — Cytokine array assay. (DOCX 10 kb) [file 13287_2016_282_MOESM3_ESM.docx]

**Cytokine Array Assay**

The cytokine profile of hlMSC-CM was determined using the Human Angiogenesis Antibody Array (R&D systems, Switzerland) following the manufacturer`s instructions. Briefly, the CM to be analysed was mixed with a cocktail of biotinylated detection antibodies. The sample/antibody mixture was then incubated with the array membrane, which was bound by its cognate immobilized capture antibody on the assay membrane. Streptavidin-horseradish peroxidase and chemiluminiscent detection reagents were added, in which a signal is produced in proportion to the amount of the analyte. The signal intensity of the cytokines was determined by chemiluminiscence detection using Versadoc (Biorad), software, Quantity One.
