# Supplementary material for: Human lung-derived mesenchymal stem cell-conditioned medium exerts in vitro antitumor effects in malignant pleural mesothelioma cell lines
Source: Stem Cell Res Ther. 2016 Feb 9;7:25. doi: 10.1186/s13287-016-0282-7 (PMC4748521; doi:10.1186/s13287-016-0282-7)
Supplement: Additional file 4: — Sphere formation. (DOCX 10 kb) [file 13287_2016_282_MOESM4_ESM.docx]

Sphere formation

Single-cell preparations of MPM cell lines were resuspended in an appropriate amount of sphere-forming medium (RPMI1640 supplemented with 20 ng/ml EGF and bFGF, [Invitrogen, Switzerland]; 4 μg/ml insulin, [Sigma, Germany]; 1 ml B27, [Invitrogen] and 1% Pen/Strep). For all cell lines, 5 x 10^3^ cells /ml /well were seeded onto a 24-well ultra-low adherent plate (Costar, USA). Cells were incubated at 37 °C, 95% humidity and 5% C0_2_ for 7–14 days. Imaging and evaluation of sphere-forming efficiency were performed on day 7. Sphere-forming efficiency (%) was determined by dividing the number of spheres formed by the original number of seeded cells. The quotient was then multiplied by 100 [11].
